# Fluence Rate-Dependent Kinetics of Light-Triggered Liposomal Doxorubicin Assessed by Quantitative Fluorescence-Based Endoscopic Probe

**DOI:** 10.3390/ijms26031212

**Published:** 2025-01-30

**Authors:** Daniel J. Rohrbach, Kevin A. Carter, Dandan Luo, Shuai Shao, Semra Aygun-Sunar, Jonathan F. Lovell, Ulas Sunar

**Affiliations:** 1 Agilent Technologies, Winooski, Vermont, VT 05404, USA; 2 Department of Biomedical Engineering, University at Buffalo, Buffalo, NY 14260, USAdandanlu@buffalo.edu (D.L.);; 3 Department of Biomedical, Engineering, Stony Brook University, Stony Brook, NY 11794, USA

**Keywords:** doxorubicin drug concentration, diffuse reflectance spectroscopy, diffuse fluorescence spectroscopy, light-triggered release, porphyrins, photodynamic therapy, endoscopic probe

## Abstract

Liposomal doxorubicin (Dox), a treatment option for recurrent ovarian cancer, often suffers from suboptimal biodistribution and efficacy, which might be addressed with precision drug delivery systems. Here, we introduce a catheter-based endoscopic probe designed for multispectral, quantitative monitoring of light-triggered drug release. This tool utilizes red-light photosensitive porphyrin−phospholipid (PoP), which is encapsulated in liposome bilayers to enhance targeted drug delivery. By integrating diffuse reflectance and fluorescence spectroscopy, our approach not only corrects for the effects of tissue optical properties but also ensures accurate drug delivery to deep-seated tumors. Preliminary results validate the probe’s effectiveness in controlled settings, highlighting its potential for future clinical adaptation. This study sets the stage for in vivo applications, enabling the exploration of next-generation treatment paradigms for the management of cancer that involve optimizing chemotherapy administration for precision and control.

## 1. Introduction

Liposomal doxorubicin (Dox) is a commonly utilized therapeutic agent for recurrent ovarian carcinoma, yet its efficacy is often hindered by inefficient drug delivery to tumor cells [1,2,3,4,5,6,7,8,9]. To address these limitations, nanocarriers such as liposomes have been developed to enhance drug distribution and efficacy. Among these, porphyrin−phospholipid (PoP) liposomes present a unique advantage, allowing controlled drug release triggered by near-infrared (NIR) light with precise spatial and temporal resolution [1,5,6,7,8,10,11,12,13,14].

This light-triggered release mechanism not only improves targeted drug delivery but also minimizes systemic side effects by confining the release to specific sites and times. For emerging nanocarriers like PoP liposomes, obtaining detailed information on the distribution of local drug concentrations is crucial for assessing therapeutic efficacy [5,15,16,17,18,19,20,21,22,23,24,25,26,27,28,29,30,31,32]. Both Dox and PoP exhibit fluorescence, which enables the use of fluorescence spectroscopy or imaging for this purpose. However, in turbid media, background optical properties can distort the raw fluorescence signal, making it challenging to accurately quantify drug concentration [22,26,33,34,35,36,37,38,39,40,41,42]. Additionally, these optical properties can attenuate the treatment light, delaying drug release.

Our porphyrin conjugate (PoP) has been through over 10 human phototherapy clinical trials. In this manuscript, we focus on PoP to assess photobleaching, which is an indicator of therapy efficacy, and to localize the drug at the local site, where doxorubicin and PoP are colocalized. Targeted drug release is expected to eliminate or reduce the side effects in the intraperitoneal cavity by protecting sensitive organs such as the intestines and liver. Here, we are investigating whether the PoP can also be used as a marker of release and ultimately as a predictor of the therapy’s efficacy, in a way similar to the way it is used in photodynamic therapy. To overcome the effect of background optical properties, quantitative fluorescence techniques are needed. 

Several spectroscopic methods have been implemented for quantification of drug concentrations in vivo [22,26,33,34,35,36,37,38,39,40,41,42]. In this study, we utilized a combination of diffuse reflectance and fluorescence spectroscopy to quantify Dox fluorescence while correcting for variations caused by absorption and scattering at both excitation and emission wavelengths. Using a catheter-based endoscopic probe, we measured the localized concentration of Dox released by NIR light in vitro and in an animal carcass. The probe, which fits into an endoscope’s working channel, provides multispectral data for Dox (~590 nm) and PoP (~680–730 nm). This approach enables precise quantification of each component in multifunctional constructs, offering significant potential for intratumoral applications in thick and deep-seated tumors.

## 2. Results

**Figure 1:**
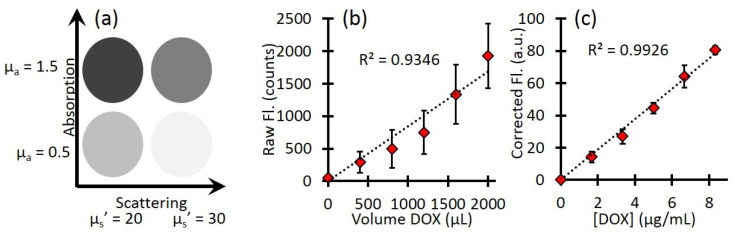
Phantom calibration. (**a**) A schematic of the phantom optical properties (**b**) Raw Dox fluorescence counts show the effect of background optical property variation. Error bars represent the standard deviation between phantoms. (**c**) Corrected fluorescence shows a linear response to increasing Dox concentration and much less variation between phantoms. Error bars represent the standard deviation between phantoms.

Calibration with free Dox provided the conversion between corrected fluorescence values and Dox concentrations, as shown in Figure 1. Figure 1a shows representative schematics of the four phantoms and their optical properties. Figure 1b shows the mean raw fluorescence signal for all four phantoms at each titration of Dox. The large error bars represent the standard deviations of the four phantoms and highlight the effects of background optical properties on the raw fluorescence. Figure 1c shows the corrected fluorescence ($C_{Dox}$ from Equation (2)). The corrected fluorescence has much smaller error bars, highlighting the improved quantification. In addition, after the correction, the slope of the line directly relates the relationship between the corrected Dox fluorescence to the true Dox concentration so that one can extract the local Dox concentration for unknown samples.

**Figure 2:**
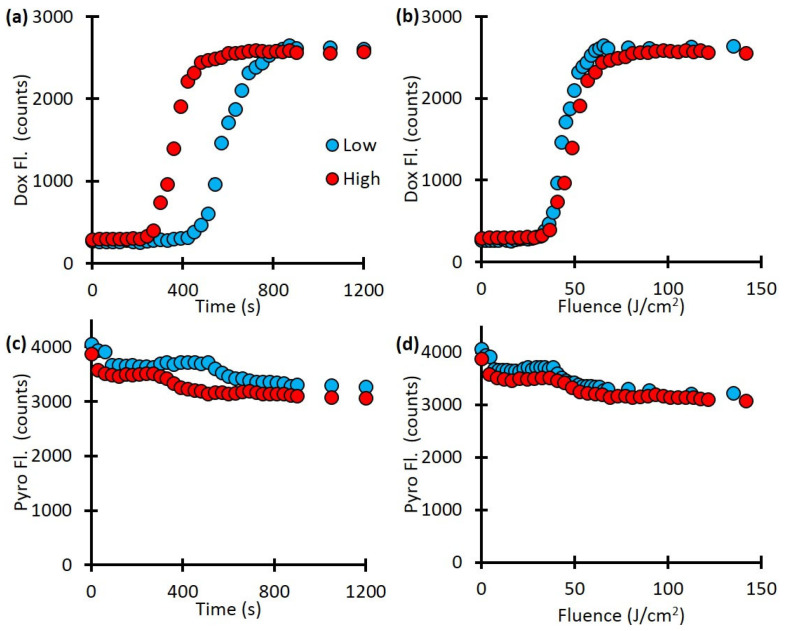
The release kinetics. The time to complete release depends on the fluence rate. (**a**) Dox release as a function of time for different fluence rates. (**b**) Dox release as a function of fluence rate. (**c**) Pyro-fluorescence signal as a function of time. (**d**) Pyro-fluorescence signal as a function of fluence rate indicates the photobleaching of the porphyrin component.

Next, we investigated the release kinetics with respect to light-irradiation fluence rate. Our fluorescence spectroscopy measurements showed a fluence-rate-dependent difference in drug-release kinetics (Figure 2a). At the lower fluence rate, Dox release started only after 450 s and was completed by 900 s, while at the higher fluence rate, Dox release started after 270 s and was completed by 510 s. This is a difference in release time: 450 seconds for the low fluence rate and 240 seconds for the high fluence rate. However, when the data are plotted as a function of light dose (mW/cm^2^), the two curves are much more similar (Figure 2b). As Figure 2c,d indicate, there was a decrease in the Pyro fluorescence as well, approximately from 4000 counts to 3000 counts, indicating the photobleaching of the porphyrin component.

**Figure 3:**
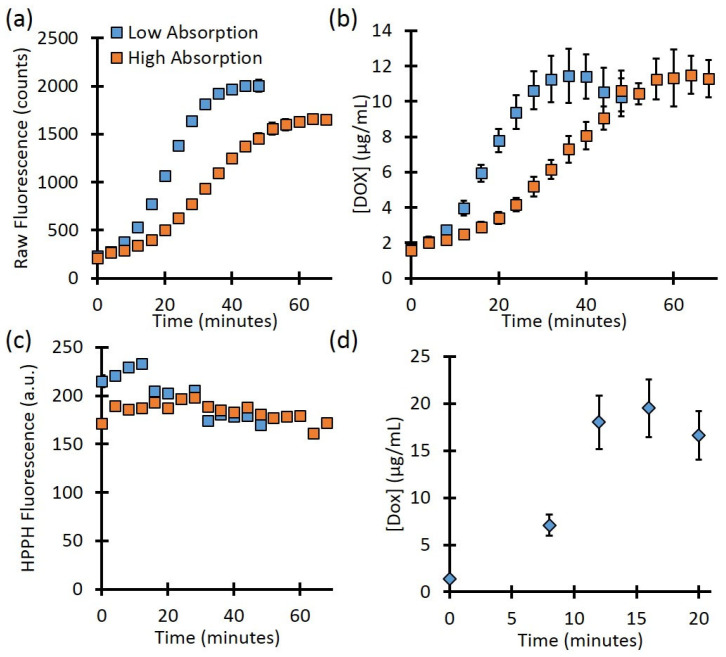
(**a**) Background absorption differences showed different final values of raw fluorescence after release. (**b**) Corrected fluorescence fitting showed the same final Dox concentration after release for the two phantoms. (**c**) Pyro (PoP, HPPH) fluorescence showed a slight decrease throughout treatment. (**d**) The release in a mouse model.

The fluence rate can be precisely controlled for optically clear phantoms in a cuvette. However, for turbid media, the true local-site fluence is unknown since the background optical properties will affect the delivered treatment light. The background optical properties will also affect the determination of fluorophore concentration. To mimic this condition, we checked the kinetics for high- and low-absorption cases. As Figure 3a shows, the raw fluorescence for phantoms with the same PoP−D liposome concentration showed different rates of release, as well as different values at full release. The phantom with lower background absorption showed a final Dox signal of 2000 ± 40 counts, while the higher-absorbance phantom showed 1700 ± 50 counts, a difference of 17.5%. However, after the Dox concentration had been quantified (Figure 3b), the full release values for the low- and high-absorbance phantoms were 11.4 ± 1.5 µg/mL vs. 11.5 ± 1.1 µg/mL, respectively, a difference of less than 1%. As Figure 3c shows, the signal from Pyro (PoP, HPPH) decreased slightly throughout treatment for both the low- and high- absorbance phantoms (26.8% and 10.4%, respectively). This decrease is likely due to the photobleaching of the Pyro.

## 3. Discussion

For the mouse experiment, the expected value of Dox concentration based on the concentration of the preparation was 22 µg/mL. The corrected fluorescence fitting determined the Dox concentration to be 19.5 µg/mL, a difference of 11.4%. This difference could be due to the partial volume effect caused by the skin layer. It is clear that Dox accumulates at the local site with time, which is the main requirement for an effective cell kill. It is interesting to note that the PoP amount was photobleached with time. This aligned well with previous observations by us and others related to photodynamic therapy (PDT) treatment of tumors with HPPH. Since the release light intensity is of the same order of magnitude as that used in PDT, in which photosensitizers (like HPPH) are photobleached due to porphyrin, oxygen, and light interaction, it would be interesting to test the combined effect (PDT and Dox-chemo) for improved cell-killing efficacy.

An important part of the fluorescence quantification is the correction for attenuation of excitation light. This was accomplished by allowing some excitation light to “leak” into the detector fiber. This leakage corresponds to the reflected excitation light and depends on the optical properties at the excitation wavelength. Kim et al. [34,37] used a similar process for fluorescence quantification, but instead of measuring the $R_{ex}$, they calculated $R_{ex}$ using knowledge of the tissue’s optical properties. For our method, it is not necessary to measure the tissue’s optical properties, which enables the fluorescence quantification to be performed very quickly. However, in cases of very high absorption or longer source−detector separations where the excitation leakage may not be seen, the calculation method will provide more reasonable estimates of $R_{ex}$. In these studies, the $R_{ex}$ signal was always seen.

In our previous work, we showed that spatial frequency domain imaging (SFDI) can quantify the release of doxorubicin [1,43,44]. While the SFDI technique is well-suited for use on superficial surfaces, it may not work for tumors located deeper inside the body. In those cases, quantitative fluorescence spectroscopy with a needle probe can be used.

However, this approach comes with several limitations. Use of a single mouse may appear too limited to allow for extrapolation to the complex biological processes of in vivo systems. This initial experiment was designed primarily to optimize our measurement system, focusing on assessing close representations of optical absorption and scattering-dependent light attenuation and variation in the fluorescence. Such preliminary tests are crucial for refining our methodologies before extensive application in live animal models. It is indeed true that using a live animal model provides a closer approximation of real conditions. We are currently developing a more comprehensive in vivo micrometastasis model and plan to perform these measurements in a more realistic setting in the near future, which will allow us to provide statistically significant data and enhance the robustness of our findings. While the catheter-based probe provides precise point measurements, it lacks the capability for spatially resolved imaging over larger areas, which could be beneficial for identifying heterogeneous drug distribution in tumors. The system’s ability to measure fluorescence at depths is limited due to limited source−detector separation (here 260 µm). We used the animal’s carcass, which may not fully replicate the heterogeneity and complexity of in vivo cases. The study focused on a specific set of light parameters (fluence rate and dose). A broader exploration of different fluence rates and dosimetry schemes is needed to establish universally applicable guidelines for light-triggered drug release. To address these limitations, future research could explore multimodal imaging approaches combining spatial resolution with the point-based probe, validate the methodology in larger and more clinically relevant animal models, and conduct pilot clinical studies to assess feasibility and efficacy in humans.

## 4. Materials and Methods

### 4.1. Drug Preparation

**Figure 4:**
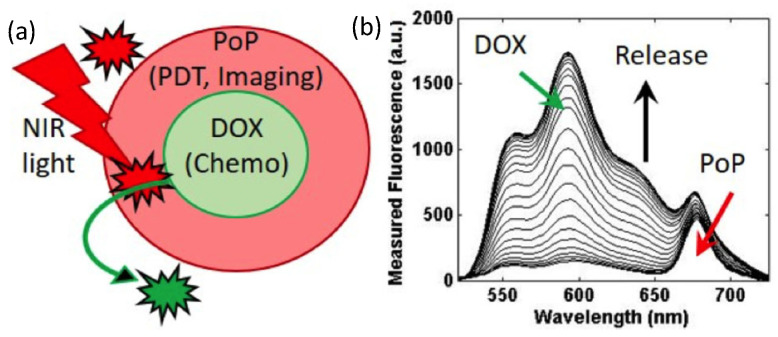
Liposomal formulation of a “nano-balloon” for imaging and treatment. (**a**) PoP red (or Dox) fluorescence can be used to localize the cancer cell. NIR light triggers release of the drug (Doxorubicin-Dox) when Dox is in the middle of the “nano-balloon”. When NIR light activates the PoP (HPPH-porphyrin), it releases the Dox. (**b**) The combined fluorescence spectra from Dox and PoP indicate that fluorescence increases during the release, which allows for an image-guided drug-delivery approach.

Our approach involves the imaging and controlled release of nano-constructs, specifically, porphyrin−phospholipid-doped (PoP-D) liposomes triggered directly by near infrared (NIR) light (Figure 4). We use the fluorescence of the PoP liposome’s outer porphyrin layer (PoP, shown in red, Figure 4a), which, prior to release, is the main imaging marker, to quantify initial drug concentration. Co-localized imaging of Dox and porphyrin fluorescence utilizes two fluorescence contrasts. Initial porphyrin fluorescence allows quantification of the maximum Dox available for release. Once Dox is released (Figure 4b), Dox fluorescence is the imaging marker used to quantify locally released Dox.

The details of our PoP−Dox (PoP−D) liposome formulation have been described elsewhere [1,10,43,44]. PoP liposomes incorporated PoP as recently reported [45]. Briefly, PoP liposomes were synthesized from pyro-lipid through esterification of pyro with lyso-C16-PC using 1-Ethyl-3-(3-dimethylaminopropyl)carbodiimide (EDC) and 4-dimethylaminopyridine (DMAP) in chloroform. The liposomes were formed by dispersion of porphyrin-lipid, PEGylated-lipid, cholesterol, and distearoylphosphatidylcholine in chloroform, and formation was followed by solvent evaporation. A 20 mg/mL lipid solution was extruded through a high-pressure lipid extruder with a 250 mM ammonium sulfate solution using sequentially stacked polycarbonate membranes of 0.2, 0.1, and 0.08 µm pore size, and the solution was passed through the extruder 10 times. Free ammonium sulfate was removed by overnight dialysis in a 10% sucrose solution with 10 mM HEPES at pH 7. Dox was loaded by incubating the liposomes at 60 °C for 1 hour, achieving a loading efficacy of over 95%, as confirmed by G-75 column tests. The self-assembly statuses and elution positions of PoP liposomes were tracked using 420 nm excitation and 670 nm emission, while Dox was detected using 480 nm excitation and 590 nm emission in a fluorescence plate reader (TECAN Safire).

### 4.2. Instrumentation

The release kinetics of doxorubicin from the PoP−D liposomes was determined using a custom fluorescence spectroscopy setup. A cuvette was placed in a fiber-coupled four-way holder (Ocean Optics) with one port connected to a 455 nm laser (Thorlabs) and a 90-degree port connected to the sensitive channel of a spectrometer (Ocean Optics). The system was controlled with a custom LabView program. For release, a 660 nm LED (Mightex) was placed above and focused into the cuvette. Measurements were acquired before PoP−D liposomes were added (PBS only) and after each round of treatment light (every 30 s for 30 min). Three measurements were acquired at each time point for averaging.

**Figure 5:**
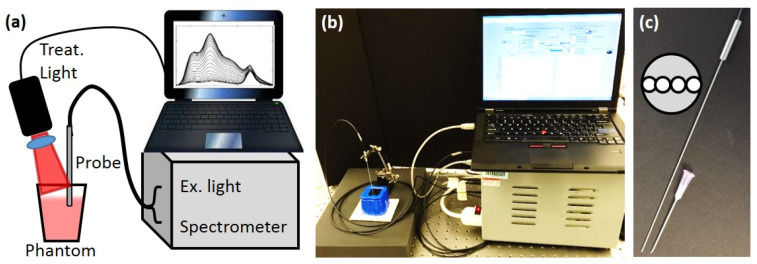
Instrument setup. (**a**). Diagram of interstitial measurement and treatment system (**b**) Picture of the setup. (**c**) The picture of the interstitial needle probe. An 18-gauge needle is shown for size comparison. The inset shows the layout of the probe face.

To better mimic the in vivo case, a custom diffuse optical spectroscopy system combining diffuse reflectance spectroscopy (DRS) and diffuse fluorescence spectroscopy (DFS) was used as shown in Figure 5a,b [28,32,44,46,47,48]. Briefly, the DRS setup consisted of a tungsten−halogen broadband white light (HL-2000-FHSA, Ocean Optics) as the source and the Master channel of the spectrometer as the detector. The light was directed to the target with one source fiber, and the diffusely reflected light was collected with one detector fiber (200 µm dia), with a source−detector separation (SD) of 260 µm. For the DFS, a 455 nm laser diode was used as the excitation source and the Slave channel of the spectrometer was used as the detector. A 500 nm long pass filter allowed measurement of the fluorescence signal (SD = 260 µm), along with a “leakage” signal of the excitation light. The reflected excitation light of the laser diode and the reflected white light from DRS were used to correct the raw DFS signal. All four fibers were contained within a narrow 18-gauge needle probe, as shown in Figure 5c.

### 4.3. Doxorubicin Quantification

A quantitative fluorescence spectroscopy model, similar to that used by Kim et al. [34,37], was used. Correction is needed to account for the attenuation of the excitation light into the sample, as well as for the attenuation of emission light out of the sample.

(1) $$FL\left(\lambda \right)=\frac{1}{R_{ex}}*\frac{f_{em}(\lambda )}{R_{em}(\lambda )}$$

**Figure 6:**
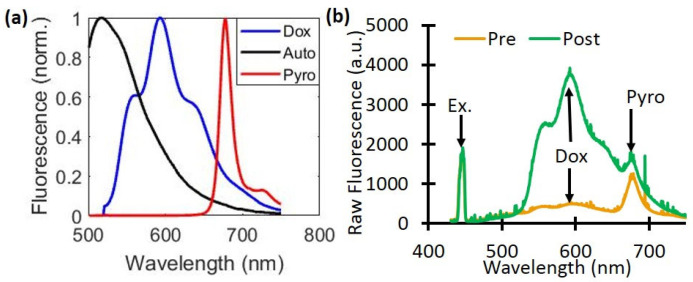
Dox and PoP (Pyro) fluorescence quantification. (**a**) The basis spectra of autofluorescence (Auto), Doxorubicin (Dox), and PoP were used to quantify fluorescence concentrations. (**b**) A representative raw fluorescence signal measured pre- and post-Dox release.

The raw fluorescence signal ($f_{em}(\lambda )$) was first divided by the white-light reflectance spectrum ($R_{em}$(λ)) and then scaled by the peak value of the excitation light leakage at 455 nm ($R_{ex}$). The corrected emission (*FL*(*λ*)) was then fit to a model of tissue fluorescence, which included background autofluorescence (*AF*), *Dox*, and the PoP liposomes (HPPH, also called Pyro), as follows:

(2) $$FL\left(\lambda \right)=C_{AF}AF\left(\lambda \right)+C_{Dox}Dox\left(\lambda \right)+C_{Pyro}Pyro\left(\lambda \right),$$

whose corresponding basis spectra are shown in Figure 6a. The value $C_{Dox}$ is then directly related to the true *Dox* concentration. Sample measurements taken pre- and post-light treatment show the changes in the raw fluorescence due to *Dox* release (Figure 6b).

### 4.4. Phantom and Animal Preparation

Phantoms were prepared using Intralipid 20% (Fresenius Kabi) for scattering and India Ink (Higgins) for absorption. Four phantoms were prepared with different combinations of optical properties: µ_a_ = 0.5 and 1.5 cm^−1^, µ_s_’ = 20 and 30 cm^−1^ at 455 nm; µ_a_ = 0.4 and 1.2 cm^−1^, µ_s_’ = 15.8 and 23.8 cm^−1^ at 590 nm. Each phantom had a total volume of 120 mL. For the calibration of Dox concentration, a stock solution of 0.5 mg/mL free Dox was used. After baseline reflectance and fluorescence measurements of each phantom, increasing volumes of free Dox were added to each (400, 800, 1200, 1600, and 2000 µL), with reflectance and fluorescence measurements acquired at each addition.

Dox release was performed in phantoms prepared as above with a scattering coefficient of µ_s_’ = 20 cm^−1^, absorption coefficients of µ_a_ = 0.5 and 1.0 cm^−1^ at 455 nm, and a total volume of 70 mL. A stock solution of 2.59 mg/mL PoP liposomes was prepared, as described in the previous section. The phantom was placed on a stir plate set to 15 rpm. Simulated interstitial measurements were acquired by placing the needle probe into the liquid phantom to acquire measurements. Fluorescence measurements were acquired before and after the addition of PoP liposomes (200 µL). The stir-plate setting was increased to 30 rpm to ensure a uniform release, and the treatment light (1.72 cm diameter spot) was turned on. Fluorescence measurements were acquired every 4 minutes.

Next, a recently sacrificed nude mouse was used to mimic in vivo measurements to check and optimize the signal. The mouse was injected subcutaneously with 50 µL of a lightly scattering medium (µ_s_’ = 5 cm^−1^ at 455 nm) containing 22 µg/mL PoP liposomes to mimic a tumor under the surface, and reflectance and fluorescence spectroscopy measurements were performed. Three measurements were acquired for pre-release baseline levels by placing the probe against the tumor-mimicking sample. The treatment light (1.0 cm diameter) was directed onto the injection site, covering it completely. Fluorescence measurements were acquired every 4 minutes to assess the release kinetics during treatment.

## 5. Conclusions

In this work, we present results from a custom-built DRS and DFS system for monitoring absolute changes in Dox and PoP concentration during light-triggered release in phantoms and in a mouse carcass. We corrected for background optical properties to obtain the true Dox concentration. Quantitative Dox concentration release kinetics showed faster release with increasing laser irradiation. These results show the potential for interstitial measurements to assess Dox release in deeply seated tumors where wide-field imaging techniques cannot reach.

## Data Availability

The data that support the findings of this study are available from the corresponding author upon reasonable request.
